# Perinatal Vertical Transmission of Chikungunya Virus in Ruili, a Town on the Border between China and Myanmar

**DOI:** 10.1007/s12250-020-00245-y

**Published:** 2020-07-16

**Authors:** Jia-Yuan Shen, Man Li, Lyu Xie, Jia-Rong Mao, Hong-Ning Zhou, Pei-Gang Wang, Jin-Yong Jiang, Jing An

**Affiliations:** 1 Yunnan Institute of Parasitic Diseases, Simao Pu′er, 665000 China; 2 Ruili People’s Hospital, Ruili, 678600 China; 3 Yunnan Provincial Key Laboratory of Vector-Borne Diseases Control and Research and Yunnan Provincial Collaborative Innovation Center for Public Health and Disease Prevention and Control (YPCICPHDPC), Simao Pu′er, 665000 China; 4 Department of Microbiology, School of Basic Medical Sciences, Capital Medical University, Beijing, 100069 China

Dear Editor,

Chikungunya virus (CHIKV), an arbovirus in the family of *Togaviridae*, genus *Alphavirus*, is transmitted by the *A. aegyptii* or *A. albopictus* mosquito, and causes disease in humans characterized by fever, rash, and arthralgia (Silva and Dermody 2017; Suhrbier 2019). It was first reported in 1953 in Tanzania, and caused only a few outbreaks and sporadic cases in Africa and Asia in last century. However, in the epidemic in 2004, CHIKV acquired mutations that conferred enhanced transmission by the *A. albopictus* mosquito (Schuffenecker *et al*. 2006). Since then, it has successively caused outbreaks in Africa, the Indian Ocean, South East Asia, the South America, and Europe (Zeller *et al.* 2016).

During the southern India Ocean epidemic in 2005, the vertical transmission of CHIKV during the perinatal period was observed in Reunion island (Lenglet *et al.* 2006). Of 160 pregnant women infected with CHIKV, 33 had viremia at the time of delivery (Lenglet *et al.* 2006). As a consequence, 16 neonates developed symptoms several days after their birth, and the risk of perinatal vertical transmission was about 48.5% (Lenglet *et al.* 2006). Two years later, as reported by a cohort study focusing on the neurocognitive function of children exposed to perinatal vertical transmission of CHIKV, 51% of 33 infected children had a global neurodevelopmental delay compared to 15% of 135 uninfected peers (Gerardin *et al.* 2014). These results indicated that perinatal vertical transmission of CHIKV resulted in a poor neurocognitive outcome (Gerardin *et al.* 2014). Thereafter, sporadic CHIKV infection through perinatal vertical transmission was observed in the Island of Curacao (van Enter *et al.* 2018), Brazil (Bandeira *et al*. 2016; Lyra *et al.* 2016), Colombia (Villamil-Gomez *et al.* 2015) and India (Kumar *et al.* 2019). In a retrospective study in Thailand and a cohort study in Colombia, however, no clinical findings suggestive of perinatal vertical transmission were observed (Laoprasopwattana *et al.* 2016; Escobar *et al.* 2017). So, how is CHIKV vertically transmitted during the perinatal period and what are the risk factors? More clinical observations are needed before these questions can be addressed.

**Fig. 1:**
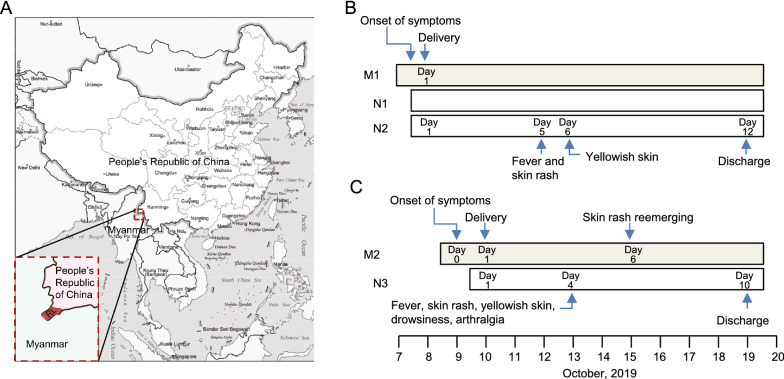
Location of Ruili and timeline of two cases in this report. **A** Ruili of Yunnan Province locates on the border between China and Myanmar. **B** and **C** Timeline of two vertical transmission cases in Ruili People’s Hospital. Three neonates (N1–N3) were infected with CHIKV through perinatal vertical transmission from a Burmese woman (M1, twin pregnancy) and a Chinese women (M2). RL, Ruili City

Ruili of Yunnan Province is a town on the border between China and Myanmar (Fig. 1A). It has the circulation of both *A. aegyptii* and *A. albopictus* mosquitoes, and is threatened by various vector-borne infectious diseases. The Ruili People’s Hospital is the largest hospital in Ruili, providing medical services to Chinese and neighboring Burmese. During September 2019, a CHIKV outbreak occurred in Ruili. By the end of December, more than 100 infected people were recorded according to the Direct Network Reporting Information System of Infectious Diseases in China. In this report, we describe two cases of perinatal vertical transmission in Ruili People’s Hospital during the 2019 CHIKV epidemic. Three neonates born to a Burmese woman (twin pregnancy) and a Chinese woman, were vertically infected with CHIKV. They all had significant perinatal complications including rash, fever, and neonatal hyperbilirubinemia but didn’t show obvious neurological symptoms. After treatment, they were all discharged from the hospital.

Case 1

A 36-year-old Burmese woman (M1) was admitted to the obstetrical department of Ruili People's Hospital because of fever and myalgia for 10 h on October 8th, 2019. CHIKV infection was suspected as a CHIKV outbreak was ongoing in the city, and was validated later by RT-PCR. The same day she was admitted to hospital, she had a C-section delivery and gave birth to 2 male neonates (N1 and N2).

The neonates were 36 weeks and 1 day gestational age, with birth weight of 2870 g (N1) and 2530 g (N2) respectively. They had neonatal asphyxia with Apgar scores of 6/6/9 (N1) and 6/6/8 (N2), respectively. They were immediately transferred to the neonatal intensive care unit (NICU) and placed on continuous positive airway pressure and later placed on BiPhasic non-invasive ventilator. Antibiotics (Cefperazone-Sulbactam 0.4 g q12h) were used to treat the presumable perinatal bacterial infection. On the fifth day of life, they developed disseminated maculopapular rash and fever with a peak of 39 °C and 38.9 °C respectively. Bathing with tepid water and taking acetaminophen orally didn’t reduce the body temperature. One day later, yellowish skin appeared and the maximum serum indirect bilirubin reached 113.1 μmol/L (N1) and 100.6 μmol/L (N2) respectively. While in hospital, no convulsion, screaming, sleepiness and other neurological symptoms were observed. CHIKV infection was confirmed by RT-PCR. The twin progressed well and were discharged from hospital on the 12th day after delivery (Fig. 1B and Table 1).

**Table 1:** Characteristics of vertically infected neonates in Ruili People’s Hospital

	M1		M2
Age	36		23
Nationality	Myanmar		China
EGA at CHIKV infection (weeks + days)	36 + 1		38 + 3
EGA at delivery (weeks + days)	36 + 1		38 + 4
C-section delivery	Yes		Yes
	N1	N2	N3
Sex	Male	Male	Female
Apgar score (1/5/10 min)	6/6/9	6/6/8	9/10/10
Birthweight (gram)	2870	2530	3080
Amniotic fluid turbidity	No	No	Yes
Asphyxia neonatorum	Yes	Yes	No
First day of fever	4th	4th	3rd
*Neonatal symptoms*			
Fever	Yes	Yes	Yes
Irritability	No	No	No
Skin rash	Yes	Yes	Yes
Yellowish skin	Yes	Yes	Yes
Drowsiness	No	No	Yes
Indirect bilirubin (μmol/L)	113.1	100.6	160.8
Length in hospital (days)	11	11	9
Death	No	No	No
RT-PCR	+	+	+

Case 2

A 23-year-old Chinese woman (M2) developed fever, chest rash, and arthralgia on October 9th, 2019 at 38 weeks and 3 days of gestation. She was admitted to the obstetrical department of Ruili People's Hospital on Oct, 10th, and had a C-section delivery the same day. Her serum was positive for CHIKV IgM. CHIKV infection was further confirmed by RT-PCR. Six days after delivery, the skin rash reemerged.

The neonate (N3) was a female with birth weight of 3080 g. Her Apgar score was 9/10/10, but the amniotic fluid was slightly stained. She was doing well until October 13th when she developed a macular erythematous rash, yellowish skin, drowsiness, arthralgia and temperature of 39 °C. The maximum of blood indirect bilirubin was 160.8 mmol/L as recorded on Oct 14th. CHIKV infection was verified with RT-PCR. On the sixth day of her life, muscle tension was recovered but arthralgia was still present. No screaming, convulsion or other nervous system symptoms were observed while in hospital. After 6 days of antipyretic (Amoxicillin, Clavulanate Potassium 0.2 mL q12h, and Cefperazone-Sulbactam 0.4 g q12h) and antiviral treatment (IFNB-1α 800,000 iu bid), she progressed favorably and was discharged on her 10th day of life (Fig. 1C and Table 1).

Because dengue virus (DENV) is circulating in this region, all febrile patients should first be tested for DENV infection, and the negative cases will then be subjected to CHIKV or ZIKV detection by RT-PCR (Primers used for CHIKV RT-PCR: F: 5′- GGGCGGGTAGTCCATGTTGTAGA-3′; R: 5′-ACCGGCGTCTACCCATTCATGT-3′). All reported CHIKV cases in this study were negative for DENV and ZIKV.

According to a systematic review and meta-analysis on perinatal vertical transmission of CHIKV, the symptoms of infected neonates include fever, irritability, hyperalgesia, diffuse limb edema, rashes and occasionally sepsis-like illness and meningoencephalitis (Contopoulos-Ioannidis *et al.* 2018). Moreover, the manifestations are usually presented within the first week of life, but not at birth (Contopoulos-Ioannidis *et al.* 2018). In our report, the pregnant women had either neonatal asphyxia or amniotic fluid turbidity during delivery and C-section delivery was thus performed, indicating that the prenatal CHIKV infection increased the risk of pregnant women and neonates. Fever and rash were observed in all neonates, which presented on day 5 (case 1) and day 4 (case 2) respectively, keeping in line with previous reports (Contopoulos-Ioannidis *et al.* 2018). Besides fever and rash, all neonates exhibited neonatal hyperbilirubinemia. Hyperbilirubinemia is a higher-than-normal level of bilirubin in the blood. Neonatal hyperbilirubinemia is usually manifested as neonatal jaundice, which is a yellowish discoloration in the eyes and skin of a newborn baby due to high bilirubin levels. In many cases there is no specific underlying disorder (physiologic). In other cases, it results from red blood cell breakdown, liver disease, infection, hypothyroidism, or metabolic disorders (pathologic). Hyperbilirubinemia during CHIKV infection has not been reported before, so that the underlying mechanism and its association with CHIKV infection need further investigation. None of the neonates had obvious neurological symptoms. However, as more than half of vertically infected children during perinatal period have been shown to have a global neurodevelopmental delay (Gerardin *et al.* 2014), appropriate follow-up is necessary to evaluate the long-term impact of CHIKV infection on neurodevelopment in this region.

These are the first cases of perinatal vertical transmission of CHIKV recorded in China. In 2010, China had a small epidemic of CHIKV in Guangdong province (Wu *et al.* 2013), which was followed by a few imported or sporadic cases in various regions in recent years. The circulation of CHIKV in Yunnan Province has been reviewed by Xia *et al.* (2018). The first imported sporadic CHIKV infection was reported in Xishuangbanna, Yunnan, in 1987, and the first isolation of CHIKV in China was taken from serum of a patient at that time (Xia *et al.* 2018). In 2001–2004, two serological investigations were carried out in Yunnan Province and the results showed that 2.63%–11.03% health people were positive for CHIKV antibody in the Lower Reaches Area of Lancang River in Yunnan Province (Wang *et al.* 2013). The circulation of CHIKV in border region between China and Myanmar, especially the detection of perinatal vertical transmission in the region is of significant concern. Research on the prevalence and prevention of CHIKV needs to be strengthened in China.

Since September 2019, more than 100 people have been infected with CHIKV according to the Direct Network Reporting Information System of Infectious Diseases in China. Except two women in our report, no other pregnant women were infected by CHIKV during the intrapartum period. Thus, in the three neonates born to two women with intrapartum infection, the risk of perinatal vertical transmission is 100%, which is similar to that observed in Reunion island, Brazil, Colombia and India (Lenglet *et al.* 2006; Villamil-Gomez *et al.* 2015; Bandeira *et al. *2016; Lyra *et al.* 2016; Kumar *et al.* 2019). In contrast, of 15 women delivered during acute CHIKV infection, as reported in 2015 in Colombia, 12 neonates were hospitalized to rule out vertical transmission, though no clinical findings suggestive of neonatal CHIKV infection were observed (Escobar *et al.* 2017). A similar result was also reported in Thailand in 2008 (Laoprasopwattana *et al.* 2016). It is still unknown why the rate of perinatal vertical transmission varied among these countries. During the epidemic in Reunion island, CHIKV acquired an A226V mutation in the envelop protein, which enhanced viral replication in *A. albopictus* mosquito (Schuffenecker *et al.* 2006). Thus, the difference of virus strain or the difference in mosquito might account for the discrepancy in these reports. To identify the factors determining perinatal vertical transmission of CHIKV, more genetic and animal studies are needed.

Thousands of neonates born with microcephaly during the epidemic of Zika virus (ZIKV) raised concerns on mother-to-child transmission of vector-borne viruses. The observation of perinatal vertical transmission of CHIKV suggested attention should be paid not only to ZIKV. Unlike ZIKV whose vertical transmission occurs during the first trimester, CHIKV is usually transmitted from mother to child at the perinatal period (Contopoulos-Ioannidis *et al.* 2018). The risk of neonatal infections was 50% among intrapartum maternal infections vs 0% among antepartum/peripartum maternal infections (Contopoulos-Ioannidis *et al.* 2018). Although the mechanism underlying perinatal vertical transmission of CHIKV is not clear, the time point makes it impossible to terminate pregnancy, so its impact could be greater than ZIKV. More attention is needed for the long-term impact of CHIKV on the health of newborns, and more efforts are required to clarify the mechanisms of pathogenesis to propose effective strategies to prevent neonatal CHIKV infection.
